# Misannotation of multiple-nucleotide variants risks misdiagnosis

**DOI:** 10.12688/wellcomeopenres.15420.2

**Published:** 2020-01-09

**Authors:** Matthew N. Wakeling, Thomas W. Laver, Kevin Colclough, Andrew Parish, Sian Ellard, Emma L. Baple

**Affiliations:** 1 Institute of Biomedical and Clinical Science, University of Exeter Medical School, Exeter, Devon, EX2 5DW, UK; 2 Molecular Genetics Department, Royal Devon and Exeter NHS Foundation Trust, Exeter, Devon, EX2 5DW, UK; 3 Clinical Genetics Department, Royal Devon and Exeter NHS Foundation Trust, Exeter, Devon, EX2 5DW, UK

**Keywords:** multi nucleotide variants, GnomAD, GATK, variant calling, next generation sequencing, genetic testing

## Abstract

Multiple Nucleotide Variants (MNVs) are miscalled by the most widely utilised next generation sequencing analysis (NGS) pipelines, presenting the potential for missing diagnoses. These variants, which should be treated as a single insertion-deletion mutation event, are commonly called as separate single nucleotide variants. This can result in misannotation, incorrect amino acid predictions and potentially false positive and false negative diagnostic results. Using simulated data and re-analysis of sequencing data from a diagnostic targeted gene panel, we demonstrate that the widely adopted pipeline, GATK best practices, results in miscalling of MNVs and that alternative tools can call these variants correctly. The adoption of calling methods that annotate MNVs correctly would present a solution for individual laboratories, however GATK best practices are the basis for important public resources such as the gnomAD database. We suggest integrating a solution into these guidelines would be the optimal approach.

## Introduction

The rapid progress and reduced cost of Next Generation Sequencing (NGS) has transformed approaches to genomic research and clinical diagnostic testing ^1^. While single-gene tests, for instance using Sanger (dideoxy) sequencing, will produce a short list of variants which can be manually evaluated, this is not feasible for next generation analysis. Sequencing at this scale requires highly automated analysis pipelines. High throughput sequencing services are dependent on automated tools to annotate and classify variants by potential consequence. For this reason, it is particularly important that any tools used to call and annotate variants do so accurately without the need for any manual assessment to avoid potential misdiagnosis.

Multiple Nucleotide Variants (MNVs) ^2^ present a particular challenge for automated NGS analysis pipelines. These variants consist of multiple Single Nucleotide Variants (SNVs) located very close together on the same strand of DNA. The Human Genome Variation Society (HGVS) guidelines state that in most circumstances, two adjacent substitutions should be classified as a single deletion-insertion mutation event, rather than two or more separate SNVs ^3^.

**Figure 1.**
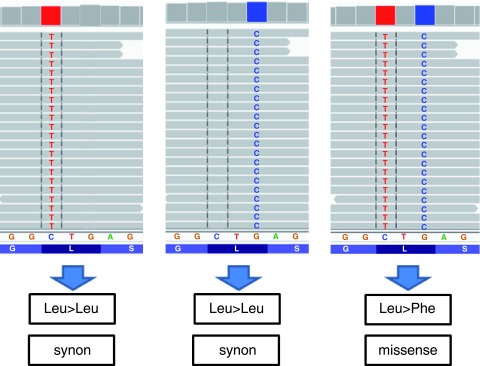
Diagram illustrating how Multiple Nucleotide Variants will be misannotated if incorrectly treated as separate variants.

MNVs that contain multiple SNVs within the same codon may have a significantly different protein consequence than if the separate SNVs are annotated independently. For instance, a CTG codon (Leu) can be changed to TTG or CTC (two separate SNVs) without any protein coding consequence, but when changed to TTC (an MNV) the consequence is a missense (see Figure 1). Importantly, some MNVs would meet the evidence criteria for pathogenicity when called as a single mutational event, but would not when each SNV is treated separately ^4^. NGS pipelines that annotate these MNVs as two independent SNVs could fail to correctly identify a pathogenic variant, potentially negatively impacting on clinical care.

Most standard NGS variant calling pipelines, including the widely adopted GATK best practices ^5^, do not deal with MNVs correctly - calling them as separate SNVs ^6^. Consequently, most laboratories using NGS technologies are at risk of miscalling these variants. Some NGS variant callers incorporate haplotype information to correctly call MNVs ^7, 8^. Another approach to correctly call MNVs is to re-process variant calls, for example using the Multi-Nucleotide Variant Annotation Corrector (MAC) ^9^. There is also a GATK tool, ReadBackedPhasing ^10^, which performs phasing of SNVs based on the overlap between reads and uses this information to call variants. However, this tool is not part of the current versions of the widely followed GATK best practice guidelines.

The scale of the potential problem with MNVs was highlighted by the ExAC database. The variants within this data set were called using a GATK best practices pipeline which does not recognise MNVs as single mutation events. Lek *et al*. ^6^ identified an average of 23 MNVs that were incorrectly annotated by the original analysis within each whole exome in the ExAC data set. In total, 778 MNVs where a STOP codon should have been annotated were noted, including multiple examples within known dominant disease genes. Crucially, they identified 10 MNVs that have previously been reported as pathogenic, but which were missed by the original pipeline. The impact of MNVs has also been highlighted by the diagnosing developmental disorders (DDD) study – Kaplanis *et al.* ^11^ showed that 2% of *de novo* variants appeared as part of MNVs and that these were significantly enriched in genes associated with developmental disorders in affected children.

HGMDpro is a commercially owned, curated database of published putative pathogenic variants associated with human genetic disorders widely used by genomic diagnostic and research laboratories. There are 628 2bp MNVs, 108 3bp MNVs and more than 150 larger MNVs listed within this database. These previously reported pathogenic variants are at risk of being inaccurately called by standard analysis pipelines. These misannotations represent potential misdiagnoses unless this problem is fully addressed.

In order to investigate the potential extent of this problem for clinical diagnostic services we devised two experiments. Firstly, to establish how MNVs are analysed we modified a set of NGS data to create simulated MNVs and processed this data using both a standard GATK best practices pipeline, and pipelines incorporating GATK ReadBackedPhasing ^10^, VarDict ^7^, Platypus ^12^ or MAC ^9^. Secondly, we re-analysed a cohort of 1447 samples previously tested using a targeted panel of genes for diagnosis of monogenic diabetes and congenital hyperinsulinism ^13^ to determine if any potential diagnoses were missed.

 By simulating MNVs in NGS sequencing data and testing for them using a typical NGS pipeline employed by an NHS diagnostic laboratory, we demonstrate that MNVs are incorrectly annotated by standard diagnostic NGS pipelines, potentially generating false positive and false negative results and negatively impacting on patient care.

## Methods

### GATK best practices pipeline

The Molecular Genetics Laboratory at the Royal Devon & Exeter NHS Foundation Trust routinely uses a targeted NGS testing pipeline to interrogate an extended panel of genes associated with monogenic diabetes and congenital hyperinsulinism ^13^. This uses GATK 3.7.0. The pipeline aligns reads to the hg19/GRCh37 human reference genome with BWA mem 0.7.15 ^14^, applies Picard 2.5.0 for duplicates removal ^15^ and GATK IndelRealigner for local re-alignment ^16^. GATK haplotypecaller is then used to identify variants and these are annotated using Alamut batch version 1.5.2 (Interactive Biosoftware, Rouen, France). This analysis approach is based on the GATK best practice guidelines ^5^. We also tested GATK 4.0.11.0 to see if the problem had been corrected in the later version of the software.

### Generating simulated MNVs

To determine whether the pipeline correctly annotates MNVs, we generated a BAM file containing five simulated MNVs in the *HNF4A* gene. These MNVs are detailed in Table 1. Each variant was generated as a homozygous call (GT 1/1 with no reads supporting the reference allele). We processed these variants with the standard GATK best practices pipeline described above.

**Table 1.** Simulated Multiple Nucleotide Variants within the *HNF4A* gene. Variants are described according to Human Genome Variation Society sequence variation nomenclature guidelines ^17^.

Variant Number	Genome position (GRCh37)	Nucleotide position	Codon position	Wild-type codon	Variant codon
1	20:43052669_43052671	NM_175914:c.838_840	p.Leu280	CTG	TTC
2	20:43053017_43053019	NM_001030004:c.1186_1188	p.*396	TAA	TGG
3	20:43056977_43056979	NM_175914:c.1066_1068	p.Ser356	TCC	AGC
4	20:43058207_43058209	NM_175914:c.1261_1263	p.Ser421	TCT	TGA
5	20:43058219_43058221	NM_175914:c.1273_1275	p.Lys425	AAG	AGT

This dataset is publicly available at https://github.com/rdemolgen/MNV-test-data to provide a simple method for laboratories to test if their current analysis pipeline will annotate MNVs correctly.

### Re-processing with alternative tools

To investigate whether using alternative tools results in correct annotation of MNVs, we re-processed the VCF file of simulated MNVs using GATK 3.6.0 ReadBackedPhasing ^10^ (default parameters plus “-maxDistMNP 2 -enableMergeToMNP”) or MAC 1.2 ^9^ then annotated the resulting VCF files using Alamut batch version 1.5.2 (Interactive Biosoftware, Rouen, France). We also tested re-calling the variants using VarDict 1.4 ^7^ and Platypus 0.8.1 ^12^.

### Investigating NGS targeted panel data for MNVs

Using the GATK ReadBackedPhasing tool ^10^, we re-examined a set of 1447 samples previously sequenced using a custom panel of genes for the diagnosis of monogenic diabetes and congenital hyperinsulinism ^13^ to determine if any MNVs with an incorrect annotation were present.

## Results

### Simulated MNVs are miscalled using GATK best practices

All five of the simulated MNVs described above were called as two separate SNVs using GATK best practices, and thus annotated incorrectly using Alamut batch version 1.5.2 (see Table 2). Variant 1 was incorrectly called as two separate synonymous variants (p.Leu280Leu), whereas the correct annotation is an in-frame deletion-insertion that results in the missense variant p.Leu280Phe. If used diagnostically this would result in a false negative result. Variant 2 alters a stop codon – when the MNV is treated correctly this results in a stop loss, however when each SNV is called separately the original stop codon is maintained presenting the potential for a false negative result. Variant 3 should result in annotation of a synonymous variant when correctly called, however GATK best practices incorrectly recognises this as two separate missense variants (p.Ser356Thr and p.Ser356Cys), which could result in a false positive testing result. When treated correctly, variant 4 should create a stop codon resulting in a nonsense variant, however it is inaccurately annotated as two variants, a missense (p.Ser421Cys) and a synonymous variant (p.Ser421Ser). Variant 5 is called as p.Lys425Arg and p.Lys425Asn, whereas it should be called as a different missense variant, p.Lys425Ser. This could result in either a false positive or a false negative result depending on the clinical interpretation of the missense variants.

**Table 2.** Simulated Multiple Nucleotide Variants within the *HNF4A* gene as annotated by GATK best practices.

Variant	Wild-type codon	Variant codon	GATK best practices annotation 1	GATK best practices annotation 2	Correct annotation	Likely implication for diagnostic testing ‡
1	CTG	TTC	c.838C>T p.Leu280Leu	c.840G>C p.Leu280Leu	c.838_840delinsTTC p.Leu280Phe	False negative result
2	TAA	TGG	p.*396*	p.*396*	p.*396Trpext*26	False negative result
3	TCC	AGC	c.1066T>A p.Ser356Thr	c.1067C>G p.Ser356Cys	c.1066_1067delinsAG p.Ser356Ser	False positive result
4	TCT	TGA	c.1262C>G p.Ser421Cys	c.1263T>A p.Ser421Ser	c.1262_1263delinsGA p.Ser421*	False negative result
5	AAG	AGT	c.1274A>G p.Lys425Arg	c.1275G>T p.Lys425Asn	c.1274_1275delinsGT p.Lys425Ser	False positive or negative result

‡Based on testing for dominant acting heterozygous, pathogenic loss of function variants.

### Simulated MNVs were correctly called using alternative software

As described above, when our simulated MNVs are called using GATK v3.7.0 best practices they are incorrectly called as two separate variants. In contrast when re-analysed using GATK ReadBackedPhasing ^10^, MAC ^9^ and Platypus ^12^ the separate SNVs are correctly merged into a single MNV in all five cases and the MNVs were correctly annotated by Alamut batch 1.5.2 as in-frame insertion-deletions. VarDict ^7^ correctly calls four variants but fails to call variant 1, which is a CTG to TTC non-consecutive change, as a single event. We also tested GATK 4.0.11.0 to see if the updated version of the software dealt with MNVs differently to older versions but the results were the same.

### Variants identified through an NGS diagnostic targeted panel are miscalled by GATK best practices

The Molecular Genetics Laboratory at the Royal Devon & Exeter NHS Foundation Trust utilises an NGS analysis pipeline based on GATK best practices. Having established, using simulated data that GATK ReadBackedPhasing ^10^ correctly called MNVs, we re-analysed 1447 samples tested on a diagnostic panel for monogenic diabetes and congenital hyperinsulinism ^13^ to examine if any MNVs had been incorrectly annotated using the GATK best practices pipeline.

On four occasions MNVs were found to have been miscalled as two separate single base substitution variants ( Table 3). In three cases the correct annotation for the MNV was a missense variant; however GATK best practices resulted in two different missense variants being called. The fourth MNV should also have been called as a missense variant, but was called as a nonsense variant and a different missense variant. In all four cases the variants were confirmed by Sanger sequencing prior to reporting, manual inspection of this data identified the correct variant call and thus the correct diagnosis was made. In the absence of Sanger sequencing confirmation these incorrect annotations have the potential to result in false positive or false negative results depending on the clinical interpretation of the missense variants.

**Table 3.** Multiple Nucleotide Variants found in the re-analysed data from the diagnostic panel to be incorrectly annotated as separate variants.

Gene	Wild-type codon	Variant codon	GATK best practices annotation 1	GATK best practices annotation 2	Correct annotation
*INSR*	GCC	TTC	p.Ala752Val	p.Ala752Ser	p.Ala752Phe
*EIF2AK3*	GAT	TCT	p.Asp615Ala	p.Asp615Tyr	p.Asp615Ser
*GCK*	GAG	AGG	p.Glu421Gly	p.Glu421Lys	p.Glu421Arg
*GCK*	TAC	CAA	p.Tyr61*	p.Tyr61His	p.Tyr61Gln

## Discussion

Using simulated MNVs and re-analysing data from a diagnostic NGS targeted gene panel test we have demonstrated that the current approach employed by most NGS variant pipelines, including GATK best practices, can result in MNVs being miscalled. There are important implications to this inaccuracy. In a diagnostic setting this could result in false positive or negative test results for patients with potentially serious consequences to an individual’s healthcare outcome. The significant number of previously published MNVs within known human disease genes that are listed within the HGMDPro database provides some context to the potential scale of this problem.

The GATK best practice guidelines ^5^ have been widely adopted and are employed in the analysis pipelines for the majority of diagnostic and research NGS facilities worldwide. Our analysis pipeline, based on GATK best practices, which is currently in use at our diagnostic laboratory, failed to correctly call our simulated MNVs and four MNVs identified by reanalysis of targeted gene panel data.

Our analysis demonstrated that in contrast to GATK best practices, alternative tools ^7, 9, 12^ are available which merge the nearby SNVs correctly into a single MNV, which is essential for correct annotation of variant consequence. There are two distinct approaches for correcting the problem, either changing the variant caller used to one such as Platypus ^12^ which calls MNVs correctly or post-process variant calls with tools such as MAC ^9^ or ReadBackedPhasing to correct the variant calls. Both solutions present problems integrating into existing pipelines. Platypus ^12^ does not produce the same quality metrics making it more challenging to integrate into an existing GATK based pipeline while ReadBackedPhasing does not maintain the quality information from the variant calls, in both cases making it difficult to filter variants by quality. Thus while a potential solution for individual laboratories to resolve this issue would be the integration of other tools within their NGS pipelines that deal with MNVs correctly this will present challenges integrating them. Additionally, this depends on laboratory awareness of this ongoing problem and the potential for patient harm that it presents.

In the current versions of the GATK best practices, phasing is performed by GATK HaplotypeCaller, so the ReadBackedPhasing software, which previously performed this role, is no longer being actively maintained. However, while HaplotypeCaller builds haplotypes we have demonstrated that it does not correctly utilise the information to call MNVs. ReadBackedPhasing calls MNVs but does not provide the quality score information for them that is produced for variants by HaplotypeCaller, which prevents them from being filtered by quality. Thus we suggest that the ideal solution would be for the features of software which enable correct calling of MNVs, namely the appropriate use of haplotype information, to be incorporated into HaplotypeCaller.

Adoption of a solution into the GATK best practices is the optimal solution as it does not require individual laboratories to be aware of the problem and adopt bespoke solutions. GATK is widely adopted for its ease of use: it provides an integrated suite of tools with inputs and outputs in standard formats, it has excellent documentation and a large user community solving shared problems.

Another important consideration to note is that publicly available online variant frequency resources such as gnomAD and ExAC are currently based on GATK best practices pipelines. These resources are critical to variant interpretation in rare genetic disorders as a key criterion for pathogenicity assignation is allele frequency ^4^. Currently MNVs are flagged, but still represented as multiple separate SNVs within gnomAD and ExAC. This means that even where laboratories make changes to their local pipeline to correctly call MNVs, their local data for these variants will be incompatible with these public resources, with allele frequency information being unavailable for those MNVs.

In summary, the issue of MNVs being miscalled by the most commonly employed NGS analysis pipelines continues to be an important issue. Although there are a number of tools available that call MNVs correctly, these are not currently being widely adopted. Addressing this issue by implementing changes within GATK best practices would have the greatest impact on prevention of misdiagnoses resulting from MNV calling inaccuracies and also importantly provide compatibility with the online public variant frequency databases that are central to current diagnostic variant classification.

## Key points

- Multi-nucleotide variants (MNVs) are misannotated by the most widely used next generation sequencing analysis pipelines
- Misannotation of MNVs can result in genetic misdiagnosis
- We suggest that individual laboratories should consider implementing alternative software to avoid misannotation of these variants
- The test data described in this manuscript has been made publicly available at https://github.com/rdemolgen/MNV-test-data so that laboratories can verify if their analysis pipeline correctly annotates multi-nucleotide variants
- We suggest that GATK best practices pipeline should implement a solution for MNV misannotation to ensure widespread adoption

## Data availability

### Underlying data

Simulated MNV data is available at https://github.com/rdemolgen/MNV-test-data

Archived simulated MNV data at time of publication: http://doi.org/10.5281/zenodo.3375579 ^18^

License: GNU General Public License v3.0

The dataset of 1447 samples previously sequenced cannot be shared due to patient confidentiality issues, as the genotype data could be used to identify individuals and so cannot be made openly available. Requests for access to the anonymised data by researchers will be considered following an application to the Genetic Beta Cell Research Bank ( https://www.diabetesgenes.org/current-research/genetic-beta-cell-research-bank/) with proposals reviewed by the Genetic Data Access Committee.
