# Supplementary material for: Misannotation of multiple-nucleotide variants risks misdiagnosis
Source: Wellcome Open Res. 2020 Jan 9;4:145. Originally published 2019 Oct 1. [Version 2] doi: 10.12688/wellcomeopenres.15420.2 (PMC6957021; doi:10.12688/wellcomeopenres.15420.2)
Supplement: Peer review history

# Peer review: Reviewer response for version 2

**DOI:** 10.21956/wellcomeopenres.17176.r37553

**Published:** 2020-01-10

**Authors:** Jaroslav Budis

**Affiliations:** 1 Geneton Ltd., Bratislava, Slovakia; 2 Comenius University, Bratislava, Slovakia; 3 Slovak Centre of Scientific and Technical Information, Bratislava, Slovakia

I would like to thank the authors for addressing my concerns about the first version of the manuscript. The updates improved the quality and reproducibility of the study.

I confirm that I have read this submission and believe that I have an appropriate level of expertise to confirm that it is of an acceptable scientific standard.

# Peer review: Reviewer response for version 1

**DOI:** 10.21956/wellcomeopenres.16863.r36790

**Published:** 2019-11-14

**Authors:** Jaroslav Budis

**Affiliations:** 1 Geneton Ltd., Bratislava, Slovakia; 2 Comenius University, Bratislava, Slovakia; 3 Slovak Centre of Scientific and Technical Information, Bratislava, Slovakia

The article presents a less known problem of misannotation of nearby genomic variants. The authors suggest alternative methods to correct misannotated variants. According to their analysis, the problem seems to affect only some variant callers (detected only in GATK). Overall, the article is well written and contains important information for the genomics labs. I also appreciate provided data sets for internal laboratory validation, as well as the proposed solutions for the problem. On the other hand, some parts of the article should be further clarified to improve its quality.

 The details of the implemented pipelines are not clear. Some tools (like Alamut) have a software version, others have detailed parameters (like ReadBackedPhaser). However, the rest of the featured tools have no execution details, so it's not clear which versions are affected. My main concern is the version of the GATK. It appears that the reported issues were found in an older version (3.?.?), according to the described methods (section GATK Pipeline for Best Practices), as IndelRealigner is no longer needed in current versions (as of 4.0.0). Therefore, it is important to identify if the problem persists or has been revised in newer releases. This is important information for genome laboratories, as the GATK upgrade should be less demanding than the implementation of an alternative software as proposed in the Key points.

 The authors should also make a clearer distinction between two approaches for correction: changing the variant caller (Vardict, Platypus) or postprocessing of called variants (MAC, ReadBackPhasing). The advantages and disadvantages of these solutions should also be discussed. 

 I disagree that Sanger sequencing has an advantage in MNV identification (Introduction). The Sanger has the advantage that fewer errors occur when calling. The problem, however, is the annotation. Therefore, it is not important if nearby SNPs were detected by NGS or Sanger sequencing.

 The authors state (Section Generating simulated MNVs) that one simulated MNV is a three-base pair event, but all five MNVs in Table 1 are two-base pair events.

I confirm that I have read this submission and believe that I have an appropriate level of expertise to confirm that it is of an acceptable scientific standard, however I have significant reservations, as outlined above.

# Response

**Published:** 2020-01-07

**Authors:** Thomas Laver

**Affiliations:** The University of Exeter, UK

We thank the reviewer for their helpful comments and have updated the manuscript accordingly in version 2. Below is a brief response to some of the queries raised.

GATK 4

 We have now tested GAKT 4.0.11.0 and found that it has the same problems calling MNVs as the previous version. We have added this information to the paper.

 We have also updated the manuscript with version numbers for other tools used.

Changing the variant caller vs postprocessing of called variants

 As the reviewer suggests we have updated the manuscript to make a clear distinction between the possible solutions to the problem.

Sanger sequencing

 One of the main differences between sanger sequencing and next generation sequencing is the throughput. Next generation sequencing produces a large number of variant calls thus the process for analysing them relies on automatic pipelines. Sanger sequencing is more manual and more targeted – likely sequencing a single gene or exon in a patient. Therefore the results will receive more manual inspection. However, the reviewer is correct that the problem is inherent to both methods. We have removed these sections from the paper.

Nomenclature of simulated MNVs

 The reviewer queried our designation of one simulated MNV as a three-base pair event. We meant that the MNV affects either end of a triplet codon and thus three base pairs. However, since this is not a necessary distinction to make we have removed the sentence to avoid confusion.

# Peer review: Reviewer response for version 1

**DOI:** 10.21956/wellcomeopenres.16863.r36617

**Published:** 2019-10-28

**Authors:** Dominic J. McMullan

**Affiliations:** 1 West Midlands Regional Genetics Laboratories, Birmingham Women’s and Children’s NHS Foundation Trust, Birmingham, UK

This article identifies an important conundrum in the application of NGS and variant calling in the clinical setting that although rare, may have profound clinical implications, particularly as Clinical labs move away from confirmatory Sanger sequencing of Single Nucleotide variants (SNVs). The article reads well and has a good diagrammatic representation of a single pathogenic MNV being represented as 2 innocuous SNVs when annotated by GATK alone. I would be interested to know whether the Broad Institute's move to using DRAGEN instead of GATK would impact on how MNVs are called in the gnomAD database. The Key Point summary is an excellent overview of the issues for non-bioinformaticians.

I confirm that I have read this submission and believe that I have an appropriate level of expertise to confirm that it is of an acceptable scientific standard.

# Response

**Published:** 2019-10-31

**Authors:** Thomas Laver

**Affiliations:** The University of Exeter, UK

Thanks for taking the time to review our paper. The reviewer makes a good point and we agree that it will be interesting to see the impact of using DRAGEN on gnomAD as it is now crucial for variant interpretation.
